# Explaining suicide attempt with personality traits of aggression and impulsivity in a high risk tribal population of India

**DOI:** 10.1371/journal.pone.0192969

**Published:** 2018-02-15

**Authors:** Piyoosh Kumar Singh, V. R. Rao

**Affiliations:** 1 Department of Psychiatry, All India Institute of Medical Sciences, New Delhi, India; 2 ICMR Emeritus Medical Scientist, Dept of Genetics, Osmania University, Hyderabad, India; 3 Honorary Research Professor, Genome Foundation, Hyderabad, India; University of Nebraska Medical Center, UNITED STATES

## Abstract

**Introduction:**

Suicide is a spectrum of behavior including suicide ideation and suicidal attempt and is undoubtedly the outcome of the interaction of several factors. The role of two main constructs of human nature, aggression and impulsivity, has been discussed broadly in relation to suicide, as endophenotypes or traits of personality, in research and in clinical practice across diagnoses. The objective of our study was to assess impulsive and aggressive behaviors among primitive people of the Idu Mishmi tribe, who are known for high suicide completer and attempter rates.

**Methods:**

The study group was comprised of 177 unrelated Idu Mishmi participants divided into two sets: 39 suicide attempters and 138 non-attempters. Data on demographic factors and details of suicide attempts were collected. Participants completed a set of instruments for assessment of aggression and impulsivity traits.

**Results:**

In the Idu Mishimi population we screened (n = 177), 22.03% of the individuals had attempted suicide, a high percentage. The suicide attempters also showed a significant sex difference: 35.9% were male and 64.10% were female (p = .002*). The suicide attempters (A) scored significantly higher than non-attempters (NA) on aggression (A = 23.93,NA = 18.46) and impulsivity (A = 75.53,NA = 71.59, with p value = 0.05). The trait impulsiveness showed a significantly higher difference (F (1, 117) = 7.274) in comparison to aggression (F (1, 117) = 2.647), suggesting a profound role of impulsiveness in suicide attempts in the Idu Mishmi population. Analysis of sub-traits of aggression and impulsivity revealed significant correlations between them. Using different models, multivariate logistic regression implied roles of gender (OR = 1.079 (0.05)) and impulsiveness (OR = 3.355 (0.013)) in suicide attempts.

**Conclusion:**

Results demonstrate that gender and impulsivity are strong risk factors for suicide attempts in the Idu Mishmi population.

## Introduction

Many attempts have been made to define the complex behavior of suicide. A number of predictors have been implicated in suicidal behavior from different social, psychological, and biological dimensions. Across cultures, the most robust findings in suicide research are the differences between genders and age groups [1]. The most dramatic increase overtime in suicide mortality rate has been observed in the third world countries India and China, due to their unique socioeconomic and behavioral patterns [2]. Current research indicates that the presence of psychopathology is probably the single most important predictor of suicide and approximately 90% of suicide cases meet criteria for at least one psychiatric abnormality [3]. The high prevalence of psychiatric disorders among suicide deaths and attempts has been observed and implicated as one of the best indicators of complex suicide behavior [4][5]. JJ Mann et al. (2003) proposed the stress-diathesis model to investigate the role of other factors over and above psychopathology [6]. Two main constructs of human nature, aggression and impulsivity, have been discussed broadly as endophenotypes or traits of personality, in relation to suicide, in research and in clinical practice across diagnoses [7, 8, 9]. Previous studies have established them as risk factors for suicide but it remains uncertain whether their effect on suicide is cumulative or independent. Current research indicates that the overlap between these constructs is robust, and they should be considered together, as a single “Impulsive-Aggression” phenotype [10]. However, other viewpoints emphasize their distinct latent dimensions [11].

Aggression refers to a wide spectrum of behaviors. It is an action intended to harm and identifies people who are predisposed to involve physical punishment, restriction, or verbal attacks like insults, threats, and sarcasm in actions or situations that are aversive or stressful [10]. It has been associated with suicidal behaviour in previous clinical, epidemiological, and family-based studies [12].

Impulsivity is a prominent construct of personality and embraces a multitude of behaviors that include responding prematurely, before considering the consequences, or action without foresight, poor planning, impaired self-regulation, sensation seeking, inhibitory control, risk taking, and preference for immediate over delayed rewards [13,14,15] that normally result in undesirable or deleterious outcomes. It is an adaptive dimension of personality and believed to stem from deficits in the self-regulation of affect, motivation arousal, working memory, and higher order cognitive functions [16]. Other abnormal behavioural manifestations of impulsivity have been found in attention deficit hyperactivity disorder (ADHD), addictions, and violent criminality, as well as antisocial personality disorder (ASPD), borderline personality disorder (BPD), and intermittent explosive disorder (IED) [17].

Studies assessing the prevalence of depressive traits have also found that attempters are more likely to be impulsive and aggressive [18]. Further, some have postulated the role of impulsivity in nonlethal suicide attempts or suicide gestures [19] and others found that the act of completed suicide is often not made impulsively [20].

The idea of an impulsive attempt, an attempt that is abrupt or that lacks planning, has been mentioned in the literature since at least the nineteenth century [21], and over the last decade research stirred by this idea has increased markedly [22]. In this line of thought, ethnographic accounts and monographs published by anthropologists highlight the presence of impulsivity and aggression, and their correlation, in indigenous people [23, 24, 25, 26, 27]. The presence of high rates of impulsivity and aggression in Indian tribes has also been discussed [28, 29, 30, 31]. Further, some ethnographers and scholars have mentioned the Idu (*Choolkatta*) as a warrior-like, aggressive, comparatively offensive, and barbaric clan of Mishmi [32, 33]. The Idu Mishmi, a Tibeto-Burman speaking tribe is the largest subgroup of Mishmi located in the Dibang Valley and Lower Dibang Valley districts of Arunachal Pradesh. It is a major sub tribe of Mishmi tribes of Arunachal Pradesh along with the Misu and Digaru Mishmi. The Idu population is distributed in about seventy-six clans and holds a distinctive identity due to their typical hairstyle, unique costumes, and the artistic patterns emblazoned on their cloths. They practice animism and souls for all activities are defined. They are mainly engaged in agriculture and its allied activities for their livelihood but the practice of hunting is still prevalent in the society.

The objective of our study was to assess personality traits, specifically impulsive and aggressive behavior, among the primitive people of the Idu Mishmi who are known for high rates of suicide completers and attempters [34, 35]. The present study could be apprehended as an attempt to observe a less-studied association between personality traits and suicide attempts in a high suicide risk solitary population. Our investigation of aggression, impulsivity, and suicide attempts in light of demographic factors will contribute knowledge to suicidology, toward a more comprehensive image of how these personality traits impact the risk of suicide across genders in the general population.

## Materials and methods

The study design of the present study is cross-sectional and the data set is comprised of 177 Idu Mishmi participants, of both sexes, aged 15–70 years (data in S1 Text), from families known to be suicide affected and un-affected, from Anini town, in the Dibang Valley district (altitude 1,968 m or 6,457 ft), Arunachal Pradesh state, India. Data were recorded using closed questionnaires and well-validated psychological tools in 30-minute-long, face to face, structured, in-depth interviews, after obtaining written consent. Participation was voluntary and no compensation was given to study participants. Demographic details including age, sex, marital status, education, and employment were collected by structured questionnaire. A psychiatric diagnosis of suicide attempts was based on the Columbia Suicide Severity Rating Scale (C-SSRS). Assessment of personality traits, aggression and impulsivity, was performed using the Modified Overt Aggression Scale (MOAS) and Barratt Impulsiveness Scale, 11th version (BIS-11) respectively. All the employed tools were used after pilot testing them in the Idu population and no modification was made to the schedules. Each participant’s responses were recorded in their preferred language (Hindi or English) by a trained anthropologist (PKS), as the population is multilingual.

The schedule C-SSRS (Columbia Suicide Severity Rating Scale) baseline Hindi/English version that was used to assess suicide attempts was developed to address inconsistencies in nomenclature and accurate identification of suicide behavior, as well as to be used in different settings [36]. Previous studies have examined the C-SSRS’s specificity, its sensitivity, and its convergent, divergent, and predictive interpreter validity. It is found to be sensitive to any change, and to be internally consistent (Cronbach’s alpha of 0.937) in various multisite studies [36, 37].

The BIS-11, which was used to assess the spectrum of impulsivity, is a 30-item gold standard measure [38]. It is designed to be a “multifaceted” measure of impulsivity including attentional, motor, and non-planning impulsiveness elucidating its biological, social-interpersonal, and cognitive-emotional dimensions. Its three subscales include cognitive, motor, and non-planning impulsiveness, where Cognitive/Attentional Impulsiveness denotes the tendency to make quick decisions, Motor Impulsiveness denotes acting without thinking, and Non-Planning Impulsiveness indicates lack of forethought. Respondents answer each item on a 4-point scale (“1 = never/rarely”, “2 = sometimes”, “3 = often”, and “4 = almost always/always”), and totals range from 0–120. Several studies, including studies conducted in India, have used the BIS-11 indifferent cultural settings [39] to explore the social significance and behavioral correlates of variability in impulsivity [40]. Published reliability coefficients for the BIS-11 total score (Cronbach’s) range from 0.72 to 0.83 [37].

Aggression assessment was performed with the widely-used, reliable, 25-item Modified Overt Aggression Scale (MOAS) [41], which is adapted and modified from the Overt Aggression Scale [42]. It provides a weekly assessment of aggressiveness and was developed to assess four forms of aggressive behavior: verbal aggression, aggression against property, auto-aggression, and physical aggression. Each dimension was measured separately on the basis of behavior in the previous15 days. Respondents were asked in a personal interview to report their behaviors over the past two weeks. Each domain was scored on a 5-point scale and total scores could range from 0 to 20, with higher scores indicating more aggression. The final score was produced by adding a single multiple of the verbal aggression score, two multiples of the aggression against property score, three multiples of the auto-aggression score, and four multiples of the physical aggression score. It also comprises important forms of aggression, like attempted suicide and intimidation. The psychometric properties of the Modified Overt Aggression Scale have been established by analyzing its inter-rater reliability and predictive power [43].

Statistical analysis included Chi-square and Student’s t test to analyze categorical and continuous variables respectively. The study population was screened for suicide attempt behaviour and the presence of a previous suicide attempt among individuals was considered for case and control classification. More explicitly, this study inspected personality traits, impulsivity and aggression, among Idu Mishmi suicide attempters and compared them to age- and sex-matched non-attempter control individuals. Sex wise interaction between sub-traits of studied variables was examined with the use of Pearson correlation coefficient. Multivariate analyses of variance (MANOVAs) were used for association analysis. Bivariate logistic regression was used to examine the joint effect of traits on suicide attempts using different models. All analyses were performed with SPSS version 16.0 (SPSS, Chicago). The study was approved by the Institutional Ethics Committee of the Department of Anthropology, University of Delhi. All the participants were informed about the study details and methods in their familiar language before giving their consent.

## Results

In the Idu Mishmi population we identified suicide attempters who had made one attempt (n = 31, 17.51%), two attempts (n = 5, 2.82%), and three attempts (n = 3, 1.69%) in their lifetime. Among attempters Hanging (n = 20, 64.52%) and consumption of pesticide (n = 7, 22.58%) were the most preferred modes of both sexes.

Table 1 presents the demographic characteristics of the presence or absence of suicide attempts among the Idu Mishmi participants. A high prevalence (22.03%) of suicide attempts is found in the population. There are no significant differences in age, marital status, or education. However, the data show a high frequency of suicide attempts in the groups who are above 19years old, married, and high school educated (studied & currently studying). The study finds significant differences using chi-square statistics between sexes (p = .002*) and occupations (p = .002*), with a high prevalence of female housewives and students in the suicide attempter’s category. However, in all interviewed female and male participants, the prevalence of suicide attempters is found to be 32.89% and 13.86% respectively.

**Table 1:** Demographic characteristics of the Idu Mishmi suicide attempters (Univariate chi sqaure (*ᵡ*^*2*^)Test analysis).

	Suicide Attempt			
	Absent		Present	
	N = 138	77.97%	N = 39	22.03%
**Sex**				
**Male (N = 101)**	87	63.00%	14	35.90%
**Female (N = 76)**	51	37.00%	25	64.10%
	**ᵡ**^**2**^	**9.145**	**Sig.**	.002*
**Age**				
**<19 (N = 37)**	29	21.00%	8	20.50%
**≥19 (N = 140)**	109	79.00%	31	79.50%
	**ᵡ**^**2**^	**0.005**	**Sig.**	**0.946**
**Marital Status**				
**Married**	78	56.50%	21	53.80%
**Unmarried/single**	60	43.50%	18	46.20%
	**ᵡ**^**2**^	**0.088**	**Sig.**	**0.766**
**Education**				
Illiterate	17	12.41%	5	12.82%
Middle	28	20.44%	11	28.21%
high school	49	35.77%	13	33.33%
Intermediate & above	43	31.39%	10	25.64%
	**ᵡ**^**2**^	**1.214**	**Sig.**	**.750**
**Occupation**				
Unemployed	21	15.22%	6	15.38%
Housewife	15	10.87%	13	33.33%
Employed	64	46.38%	8	20.51%
Student	38	27.54%	12	30.77%
	**ᵡ**^**2**^	**14.813**	**Sig.**	0.002*

* p value<0.05

The mean ± SD of scores on tests for personality traits, like aggression and impulsivity, in each subcategory among suicide attempters and non-attempters, sex wise, are depicted in Table 2. Significant differences are found between all suicide attempters and non-attempters in Total Aggression, and female attempters and non-attempters differ significantly in Auto-aggression. The BIS-11 produced impulsivity score analysis also shows a significant difference between suicide attempters and non-attempters in Total Impulsivity and in subcategories Non-planning and Motor impulsivity. Whereas, in sex-based analysis, only males show significant differences in the subcategoriesmotor and cognitive impulsiveness.

**Table 2:** Status of aggression and impulsivity among Idu Mishmi suicide attempters in males and females (Univariate t Test analysis).

**Aggression**									
	**Male (N = 76)**			**Female (N = 58)**			**Total (N = 134)**		
	**Present (N = 11)**	**Absent (N = 65)**	**t Test**	**Present (N = 18)**	**Absent (N = 40)**	**t Test**	**Present (N = 29)**	**Absent (N = 105)**	**t Test**
	**Mean** ± **SD**	**Mean** ± **SD**		**Mean** ± **SD**	**Mean** ± **SD**		**Mean** ± **SD**	**Mean** ± **SD**	
**Verbal aggression**	2.45±1.21	2.58±1.48	0.318	3±1.78	2.85±1.72	0.300	2.79±1.59	2.69±1.57	0.323
**Aggression against property**	2.36±1.91	1.45±1.82	1.482	1.56±1.53	1.25±1.55	0.686	1.86±1.73	1.37±1.72	1.356
**Auto-aggression**	1.55±2.54	1.06 ±1.71	0.608	1.28±1.21	0.45±0.88	2.503*	1.38±1.82	0.83±1.48	1.498
**Physical aggression**	3.64±2.66	2.69±2.12	1.12	3.33±1.75	2.58±1.82	1.507	3.45±2.10	2.65±2.00	1.837
**Total Aggression**	25.18±16.14	19.4±13.46	1.124	23.17±10.04	16.92±8.65	2.283*	23.93±12.46	18.46±11.87	2.116*
**Impulsivity**									
	**Male (N = 79)**			**Female (N = 56)**			**Total (N = 134)**		
	**Present (N = 10)**	**Absent (N = 69)**	**t Test**	**Present (N = 19)**	**Absent (N = 37)**	**t Test**	**Present (N = 29)**	**Absent (N = 105)**	**t Test**
**Non-Planning**	29.8±3.68	27.9±5.15	1.443	30.42±2.87	29.49±3.88	1.019	30.42±2.87	28.72±3.91	2.137*
**Motor**	26.1±5.15	23.23±4.81	2.302*	25.63±4.67	24.22±3.80	1.141	25.63±4.67	23.8±3.87	2.076*
**Cognitive/ Attentional**	20±1.63	18.42±3.76	2.425*	19.47±2.61	19.57±2.29	*0*.*133*	19.47±2.61	19±2.81	1.289
**Total Impulsivity**	75.9±7.11	69.55±11.14	1.746	75.53±6.13	73.46±6.33	1.181	75.53±6.13	71.59±7.06	2.973*

* p value<0.05

A Pearson’s coefficient of correlation analysis, between all subcategories and total impulsivity and aggression scores, shows high intra-correlation between traits of aggression and impulsivity in both sexes, whereas inter-correlation between categories of aggression and impulsivity is only observed in males (Table 3). In all screened males, non-planning and cognitive impulsiveness is found to be correlated with auto-aggression, motor impulsivity is correlated with physical aggression, and both total aggression and total impulsivity establish a correlation with aggression against property, physical aggression, and total aggression. In sex wise analysis, males show better correlation among the subcategories of aggression, whereas, in females, we record some negative correlation and could not find any significant correlation between the subcategories of impulsivity. The results imply that the traits of impulsivity and aggression are deeply correlated among males.

**Table 3:** Inter- correlations among traits of aggression and impulsivity in males and females of Idu Mishmi suicide attempters (Univariate Pearson correlation analysis).

**Categories /Subcategories**		**Verbal aggression**	**Aggression against property**	**Auto aggression**	**Physical aggression**	**Total Aggression**	**Non-Planning**	**Motor**	**Cognitive/ Attentional**	**Total Impulsivity**
			**MALE**							
**Verbal Aggression**	**Pearson Correlation**		0.099	0.021	.340**	.393**	0.059	-0.004	0.164	0.095
	**N**		58	58	58	58	50	50	50	50
**Aggression against property**	**Pearson Correlation**	.287*		.272*	0.111	.517**	0.191	-0.126	0.058	0.047
	**N**	76		58	58	58	50	50	50	50
**Auto aggression**	**Pearson Correlation**	.233*	.357**		0.123	.457**	0.03	0.06	0.071	0.082
	**N**	76	76		58	58	50	50	50	50
**Physical aggression**	**Pearson Correlation**	.274*	.364**	.336**		.832**	0.15	-0.063	0.102	0.081
	**N**	76	76	76		58	50	50	50	50
**Total Aggression**	**Pearson Correlation**	.386**	.675**	.680**	.842**		0.157	-0.024	0.202	0.15
	**N**	76	76	76	76		50	50	50	50
**Non-Planning**	**Pearson Correlation**	-0.072	0.187	.267*	0.193	0.232		0.092	-0.032	.566**
	**N**	71	71	71	71	71		56	56	56
**Motor**	**Pearson Correlation**	0.028	0.227	0.073	.252*	.242*	.401**		0.205	.759**
	**N**	71	71	71	71	71	79		56	56
**Cognitive/ Attentional**	**Pearson Correlation**	0.137	0.208	.240*	0.127	0.23	.541**	.488**		.517**
	**N**	71	71	71	71	71	79	79		56
**Total Impulsivity**	**Pearson Correlation**	0.024	.255*	0.233	.243*	.290*	.819**	.797**	.799**	
	**N**	71	71	71	71	71	79	79	79	
	**FEMALE**									

**. Correlation is significant at the 0.01 level (2-tailed).

*. Correlation is significant at the 0.05 level (2-tailed).

Table 4 shows an association analysis using Multivariate analysis of variance (MANOVA) between and among personality factors and the outcome of suicide attempts in the Idu Mishmi population. A one-way between-groups multivariate analysis of variance was performed to investigate the relationship between suicide attempts and personality traits. Two dependent variables were used: aggression and impulsiveness. The independent variable was suicide attempt. Preliminary assumption testing was conducted to check for normality, linearity, univariate and multivariate outliers, homogeneity of variance and covariance matrices, and multicollinearity, with no serious violations noted.

**Table 4:** Association analysis between among personality factors and outcome of suicide attempt multivariate analyses of variance (MANOVA analysis).

	Wilks' Lambda	F	Hypothesis df	Error df	Sig.	Partial eta squared
**Cumulative effect**						
**Aggression Impulsiveness (Combined)**	.934	**4.102**	**2.000**	**116.00**	.019*	**0.66**
**Individual Effect**						
**Aggression**		2.647	1	117	.106	0.22
**Impulsiveness**		**7.274**	**1**	**117**	.008*	**0.59**

* p value <0.05

There was a statistically significant difference between suicide attempters and non-attempters on the combined dependent variables, F (2, 116) = 4.102, p = 0.019; Wilks’ Lambda = 0.934; partial eta squared = 0.66. When the results for dependent variables were considered separately, the only difference reached to statistical significance, using a Bonferroni adjusted alpha level of 0.025, was impulsiveness, F (1, 117) = 7.274, p = 0.008, partial eta squared = 0.59. The significant value of impulsiveness implies the profound role of impulsiveness more than aggression, in suicide attempts in the Idu Mishmi population of the Dibang Valley.

Table 5 depicts the results of three separate regression models with potential predictors of suicide attempts. In Model1, aggression, Impulsiveness, gender, and age were analyzed separately in association with suicide attempts and the results establish separate roles of aggression, impulsiveness and gender in suicide attempts. In Model2, when only aggression, Impulsiveness, and suicide attempts were entered simultaneously to control for the impact of gender and age, Impulsiveness remained significant. Whereas in Model 3, demographic variables like gender and age were entered as independent correlates along with aggression and Impulsiveness, and we find a significant role of gender (adjusted Odds Ratio = 3.355 (0.013)* Confidence Interval = 1.289–8.738)and Impulsiveness (adjusted Odds Ratio = 1.079 (0.05)* Confidence Interval = 1.000–1.164)in suicide attempts. Although Impulsiveness remained significantly associated with an increased likelihood of suicide attempts, gender (being female) contributed significantly to the relationship.

**Table 5:** Association between gender, age, personality factors with suicide attempt among Idu Mishmi (Multivariate logistic regression analysis).

	Model-1	Model-2*	Model -3*
	Unadjusted Odds Ratio (Sig.) 95%CI	Adjusted Odds Ratio (Sig.) 95%CI	Adjusted Odds Ratio (Sig.) 95%CI
Aggression	1.044 (0.017)* 1.008–1.082	1.019 (0.348) 0.980–1.060	1.026 (0235) 0.983–1.072
Impulsiveness	1.091 (0.008)* 1.024–1.164	1.087 (0.023)* 1.011–1.167	1.079 (0.05)* 1.000–1.164
Gender 1 = Female	3.046 (0.003)* 1.453–6.384		**3.355 (0.013)** * 1.289–8738
Age 1 = 19 and above	1.030 (0.946) 0.428–2.482		0.838 (0.816) 0.190–3.708

Adjusted variables in Model 2 = Impulsiveness, Aggression. Adjusted variables in Model 3 = Gender, age, Impulsiveness, Aggression

* p value <0.05

## Discussion

This study reports a high rate of suicide attempts (22.03%) in the Idu Mishmi tribe of the Dibang Valley district of Arunachal Pradesh, India. The issue of suicide in the Idu Mishmi came to light with media reporting of suicide deaths in the Dibang Valley and Lower Dibang Valley, and was later validated by a scientific study in which 218 cases of suicide were reported, over four decades, in this tiny tribe numbering 15,000 [44]. Further, our group has published the presence of high suicide attempt rates and psychiatric traits like depression, anxiety, and eating disorders among Idu Mishmi school children and family members in the lower Dibang district of Arunachal Pradesh [34]. Ethnographic, anthropological, and sociological accounts of indigenous people from India and around the globe have posited the frequent presence of suicide behavior among primitive people [30, 31 45, 46, 47]. Several attempts have been made to unmask the causative and risk factors like psychiatric abnormality, positive family history, alcoholism, depression, having a friend who has attempted suicide, history of physical abuse and sexual abuse, female sex, etc. [48,49]. The stress-diathesis model, which includes the role of childhood trauma and personality traits like impulsivity and aggression, is another attempt to illuminate the complex behavior of suicide [4]. The population aggression score mean recorded in the Idu Mishmi population was 21.20. In a sex wise comparison, the male sex recorded a high mean value. In females, auto-aggression was found to be high. Research indicates that females were less likely to opt for direct physical violence and they can express their aggression though non-physical behaviour. Males, are quicker to show physical aggression and aggression against property. The outcome of this study, the significant differences shown between suicide attempters and non-attempters in the total aggression category, is validated by the shared neurobiology that has been proposed for suicide and aggressive behavior [8]. Aggression has been observed to be concomitant with lowered serotonin-mediated brain activity, and a pattern of emotional dysregulation in the context of interpersonal difficulties and other stressful life events, all of which can result in suicide [10, 50].

The impulsivity trait evaluation among the Idu Mishmi tribal population recorded it to be quite high with a mean of 73.56, compared with other population studies in India [51] which have recorded means of 61.71 in rural and 62.65 in urban samples. Sex wise comparison in the total population shows elevated female impulsivity, with an average 74.5 score, while males mean score was 72.73. However in the suicide attempter category, males’ impulsivity scores were found to be high. Global studies support the higher Impulsiveness scores found in females [40]. However, findings from other indigenous population based studies support the notion of high impulsivity in indigenous people. Doyle et al. (2015) recorded that 42% of non-indigenous inmates and 53% of indigenous inmates screened positive for impulsive personality [52]. A Commission for Children and Young People and Child Guardian Queensland (2009) report noted that suicide impulsivity is frequently reported more among aboriginal youth than the wider Australian youth population [53]. Impulsiveness symptoms have been implicated across neuropsychiatric disorders, with important consequences for everyday activity and quality of life. Studies have explained the variability in impulsive behaviors as stemming from genetic or temperamental roots that interact with psychological and environmental experiences [54, 55, 56,57]. The present population study was an attempt to substantiate earlier findings. Simon et al. (1994) reported that 24% of near-lethal suicide attempt survivors had thought about their suicide attempt for less than 5 minutes [58]. The present study further reports the interplay between categories and subcategories of aggression and impulsivity. Some recent studies have suggested the interplay of aggressive and impulsive behavior to be the underlying link between family history of suicide committers and new suicide attempts [59, 60]. Menon et al., 2015 listed causes of suicide death among Indians and found males’ modes differed from those of females, which could also be explained by the differences in personality characteristics [1].

The outcome of our study suggests that impulsivity and gender (being female) may contribute significantly as risk factors for suicide attempts with variability in the category and subcategories of aggression and impulsivity across sexes. Kumar et al. 2013 also observed a difference between male and female suicide attempters with respect to concurrent diagnoses, mode of attempt, and stressful life situations encountered [2]. To summarize, it can be stated that in the stress-diathesis model, aggression and impulsivity are important components of the diathesis for suicidal behavior. Inference from this study suggests that suicide behaviour needs multidisciplinary understanding and it should involve knowledge from psychologists, anthropologists, and ethnographers. The magnitude of socio-cultural factors in the cultural personality background should be studied so that a sound suicide prevention program can be planned.

## Conclusion

The present study among the Idu Mishmi, an isolated endogamous tribal group, focuses on the presence of a high suicide attempt rate and sex variability. High scores on measures of aggression and impulsivity traits were recorded, with significant differences among suicide attempters and non-attempters. Male attempters were more at risk of impulsivity, whereas females showed greater risk for aggression, but in the total studied population, the suicide risk was almost three times higher with higher aggression, and almost twice as high with higher impulsivity. The subcategories of impulsivity were deeply correlated among themselves and with subcategories of aggression. Most of the previous studies emphasized considering suicide attempts to be the best diagnostic trait of suicidal behavior [61] and that it should possess the qualities of being self-initiated, potentially injurious behavior with the presence of intent to die and nonfatal outcome [62]. This study validates a suspicion raised by earlier scholars, as the high intensity of aggression and impulsivity in indigenous populations and their association with suicide behavior was re-witnessed in the present population-specific study.

## Limitations

The limitation of small sample size is due to the small population size, low density, and difficult terrain of their habitation. We could not observe the interplay of psychiatric abnormalities and the traits of aggression or impulsivity due to the absence of a psychiatric clinic in the study area. Further, deaths due to suicide could not be quantified in the absence of postmortem based data.

## Supporting information

S1 Text Idu Mishmi Dataset. (XLSX)
